# What puzzle are you in?

**DOI:** 10.1186/s13059-022-02748-1

**Published:** 2022-08-25

**Authors:** Itai Yanai, Martin J. Lercher

**Affiliations:** 1 Institute for Computational Medicine, NYU Langone Health, New York, NY 10016 USA; 2 Institute for Computer Science & Department of Biology, Heinrich Heine University, 40225 Düsseldorf, Germany

We shall not cease from exploration

And the end of all our exploring

Will be to arrive where we started

And know the place for the first time

– T.S. Eliot

Nature is a tapestry of puzzles, and solving them is a central source of joy in research. Despite their complexity, nature’s puzzles can be classified in the same way as puzzles humans invented for entertainment: jigsaw puzzles, logical puzzles, puzzles where we need to find connections to phenomena outside the problem description, and puzzles that require us to think outside the box, often by identifying and dropping implicit assumptions. These archetypes can be distinguished along with two dimensions: whether they are closed-world or open-world and whether the solutions require either making connections or deeper insights into the problem structure. Solving artificial puzzles can be an important practice for the development of scientific creativity—and that is exactly what degree programs impose on their students, particularly in mathematics, physics, and engineering.

But nature’s puzzles are different from artificial puzzles in one crucial aspect: in the middle of an ongoing research project, you can never be sure what kind of a puzzle you are in. What you mistake for a complex jigsaw puzzle, where all you need to do is put the pieces in front of you into the right arrangement, may in fact be a puzzle you can only solve by identifying a connection to a different field. In research, you thus not only solve a puzzle itself, but also the corresponding meta-puzzle: what type of puzzle are you in? Being conscious of this hierarchical problem structure and switching between perspectives appropriate for different puzzle classes can boost our scientific creativity, accelerating our search for discoveries.

## Four kinds of puzzles

Doing science—as opposed to learning about science—requires creative problem solving, which we have previously discussed as “night science” [1]. We solve problems when we figure out what experiment or analysis to carry out or when we try to make sense of observations and data. Doing this is akin to solving puzzles created for amusement, such as brain teasers or jigsaw puzzles, putting us in a very similar mental space. Such artificial puzzles form a microcosm of problem-solving. Working on them, we experience a fundamental asymmetry: they seem very difficult or even impossible while we are looking for the solution; but once we know the solution, it seems almost obvious.

**Fig. 1:**
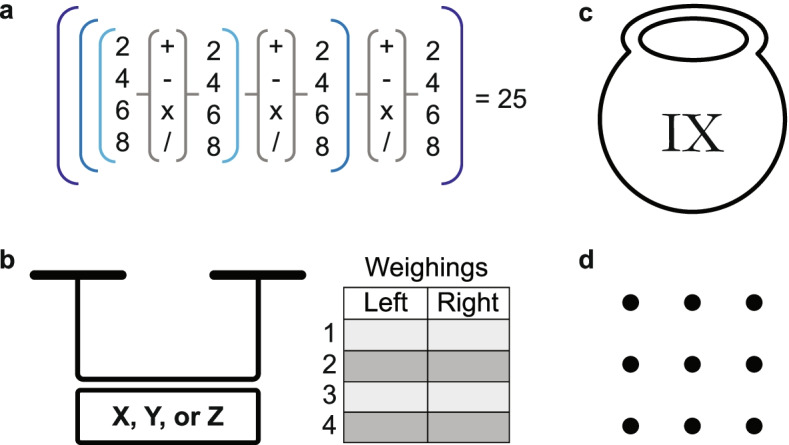
Four puzzles. **a** A mathematical jigsaw puzzle (Class I): How can you combine each of the numbers 2, 4, 6, and 8 with three different operations out of +, −, *, and / to arrive at the number 25? **b** A logical puzzle (Class II): This scale outputs one of three symbols, corresponding to “left is heavier,” “equal weights,” and “right is heavier.” While you can distinguish the symbols, you do not know their meaning. Find the four weighings that allow you to distinguish among 12 coins the one that is either slightly heavier or lighter than the rest. **c** A Class III puzzle: Add a mark to indicate that 3 of the meatballs have been eaten. **d** A Class IV puzzle: Can you connect the dots in a connected line with only 4 straight edges?

Puzzles come in different flavors, each with their own premises that circumscribe the type of the expected solution. In the conceptually simplest class of puzzles, you are presented with all of the pieces and the possible types of connections—all you need to do is to figure out how they fit together. The archetype of this class is the jigsaw puzzle, where your solution efforts are rewarded through the global image that emerges from connecting the pieces locally. As an example of a mathematical jigsaw puzzle, consider the following (also shown in Fig. 1a):

How can you obtain the number 25 by combining all of the numbers 2, 4, 6, and 8 with three different operations out of +, −, *, and /?

It is possible to solve this puzzle by brute force, trying out all possible combinations—though there are many. As the scale of such puzzles increases, so does their complexity.

In the second class of puzzles, the parts are also clearly defined, but a logical leap is required to arrive at the solution. Such “logical puzzles”—often called brain-teasers—pose a well-defined problem, the solution of which frequently involves the use of mathematical tricks. For example, consider the following logical riddle (Fig. 1b):

*Imagine you have 12 coins, 11 of which have the same weight. The remaining coin is either heavier or lighter than the rest. Can you find the odd coin with only 4 weighings? You have to use a “unique” digital balance scale that compares two weights. It outputs one of three symbols, corresponding to the following: “left is heavier,” “equal weights,” and “right is heavier.” You can distinguish the three symbols, but you do not know their meaning.*

While finding the solution is not easy, its general structure is clear: in the first weighing, you put *n* coins on the left and *n′* other coins on the right, and so forth for the three other weighings. That is all there is—there can be no tricks, such as messaging the scale’s inventor or melting the coins. What is the precise logic of choosing the solution? If we were to provide you with the solution immediately, it might seem straightforward. But trying to solve it, you will appreciate that it is not. What will be required is to shed light on the logical structure of the problem—a simplifying insight that makes the solution possible.

These first two classes of puzzles can be considered closed-world: the constituents of the solution and their possible connections are known in advance; the challenge is to assemble them in a meaningful way. In contrast, other classes of puzzles are open-world. Here, the answer is not inscribed within a closed box—you are missing important information on the components or the structure of the solution. Hence, in the third class of puzzles, you need to make a connection to a realm external to the problem formulation. Consider the following (Fig. 1c):

*A man cooks nine meatballs for his ailing father. He gives them to his daughter to bring to her grandfather. To make sure that all meatballs arrive uneaten, he labels the pot containing the meatballs with the roman numerals “IX,” using a permanent marker. On the way, the girl eats three of the meatballs. She has the marker, though, and while she cannot erase her father’s mark, she can add something to it. What should she do so that her grandfather does not suspect that anything is amiss?*

You might realize soon that it is impossible to get to a smaller number than 9 when staying within the roman numeral system. Thus, this puzzle requires you to connect the aspects presented in the puzzle description to something external to it. While the connection may be a simple one, finding it is complicated by the vast size of the search space.

Finally, in the fourth class of puzzles, one does not need to connect to a whole other world, but rather look outside the box. You need a deeper insight that requires a mental leap—a trick! Typically, this requires you to drop a constraint on the search space that was not part of the problem description, but that you added implicitly. Consider the puzzle of the nine dots shown in Fig. 1d, which you might have seen before:

*Can you connect the dots with an uninterrupted line consisting of 4 straight edges?*

Finding the solution using five connected lines is quite simple. If you are like most people faced with this problem for the first time, you may unconsciously restrict your lines to the virtual box circumscribing the outermost dots. We can find the solution only when we drop that implicit assumption and allow ourselves to think outside of this box. It is interesting to note that the solutions to such puzzles often make us laugh, because of the startle we feel when realizing an unexpected, alternative way to see the same set of facts.

These four puzzle archetypes can be arranged on a two-dimensional grid (Table 1). The first dimension concerns the completeness of the problem formulation: closed-world (Classes I and II) versus open-world (Classes III and IV). The second dimension concerns the type of insight required: finding connections (Classes I and III) versus the need to reframe the problem—either through an insight into the problem structure (Class II) or by moving our thinking out of the box (Class IV). If you know where you are on this grid, you know which type of puzzle you are in.

**Table 1:** The 4 Classes of puzzles

	Finding connections	Reframing
**Closed-world**	Class I: jigsaw puzzles	Class II: logical puzzles
**Open-world**	Class III: outside connections	Class IV: out-of-the-box

## Research as puzzle solving

Looking at specific scientific discoveries, we can see that many fit neatly into one of the four classes of puzzles. Anything that requires following an established protocol—such as obtaining the 3-D structure of a protein, assembling a complete genome, or defining the embryonic cell lineage of an organism—is a Class I puzzle. The scientists involved know that there is a solution, and they know what ingredients it has. The ingredients “just” have to be put together in the right way. Consider the production of an atlas of cell types and states using transcriptomics (single-cell and spatial). For a given organ or an entire organism, numerical representations of the cells (or pieces) can be assembled to reconstruct the system [2]. Technical obstacles must be overcome, and new aspects may be discovered, but they are confined to the world established by the parts.

As an example of a closed-world, logical Class II puzzle, consider how Crick and colleagues, in the years that followed the discovery of the double helix, thought about what the structure of the genetic code might be [3]. Working with the constraints that the 20  naturally occurring amino acids must be encoded by only four nucleotides, they had the insight that the coding might operate in a “comma-free” fashion: “This paper deals with a mathematical problem which arose in connection with protein synthesis. We present the solution here because it gives the ‘magic number’ 20, so that our answer may perhaps be of biological significance.” Studying the problem as a logical puzzle, Crick and colleagues noticed that the set of 64 triplets (today’s “codons”) collapses to 20 if the constraint is added that it should be immediately clear in which frame the code is to be read. The four same-letter codons (“AAA,” “CCC,” “GGG,” and “TTT”) are eliminated, as using these would lead to unclear frame encoding in the case of repeats. The remaining 60 triplets fall into twenty groups, each containing three “triplets” that are cyclic permutations of each other, such as “ACG,” “CGA,” and “GAC.” If only one triplet from each set is used and the rest is avoided, there would be at most 20 usable triplets—the precise number of amino acids. Crick et al. showed that multiple sets of 20 such triplets exist that indeed make the frame unambiguous. This is a great use of logic—although, of course, it did not end up being the correct solution. But one very good idea came from it: they predicted the existence of an adaptor molecule, which was later discovered to be transfer RNA.

Much of scientific progress comes from making connections, thus constituting Class III puzzles. For example, following others, Gödel examined the notion of making a complete, contradiction-free formal system of all mathematical theorems. He found that by connecting this question to number theory, one could show the existence of statements that are neither provable nor disprovable in such formal systems, thereby demonstrating that the then-popular search for a unified and complete mathematical system was futile [4]. In a second example, Darwin famously connected the local adaptations he saw on his world trip on the Beagle with the work of economist Malthus. Malthus had pessimistically stated that since unchecked population growth would be exponential, war and disease must be a constant aspect of the human condition. Darwin realized that in this situation, the better-adapted individuals have a better chance of leaving offspring. He termed the corresponding process natural selection and proposed that it explains how adaptation in biological populations occurs over eons [5].

Some of the most surprising discoveries, though, come from solving Class IV puzzles. When a scientific problem seems impossible to solve, it may be that a bad assumption—either implicit or explicit—is constraining our search space. In these instances, we need to think outside the box. A striking example is the discovery of the function of Clustered Regularly Interspaced Short Palindromic Repeats (CRISPR). In the year 2000, Mojica and colleagues made a puzzling observation: previously observed sets of short genomic repeats, separated by equally short spacers, were not a strange anomaly of a few isolated species, but were instead widespread across prokaryotes [NEW REFERENCE: Mojica FJ, Díez-Villaseñor C, Soria E, Juez G. Biological significance of a family of regularly spaced repeats in the genomes of Archaea, Bacteria and mitochondria. Molecular Microbiology. 2000;36: 244–246. ]. How did these repeats arise? Were they selfish elements of some form, or did they perform any functions useful for the bacteria? The sequences of the repeats resisted all attempts to decipher their origin or function. So what was the bad assumption constraining the search space? Researchers around the world had dismissed the spacers between the repeats, which seemed to have no conspicuous properties, as unimportant. As it turned out, the spacers instead carry the functionally important information. They form the basis of an adaptive bacterial defense system, matching the sequences of threats such as viruses [6].

## Science is a meta-puzzle

Problem solving is deeply integrated into the formal training of scientists. Well-designed courses in undergraduate education challenge students with artificial, puzzle-like problems. However, when presented with such educational puzzles, you usually know what puzzle class you are in, just as you do when presented with puzzles for entertainment—a jigsaw is a jigsaw puzzle, and a logical puzzle is logical, no tricks allowed. Almost always, these will be closed-world puzzles, as exercise questions will be based on the facts and methods you learned in the previous weeks.

Similarly, when viewing a scientific research project in retrospect, it seems to clearly fit into a particular class of puzzles. But such simple classification derives from the benefit of hindsight—doing science in real time is different. When we actively work on a scientific problem, we have no way to be certain what kind of a puzzle we are in, or if the puzzle as we see it even has a solution. Solving research puzzles is a hierarchical problem. You not only have to find the solution to a puzzle that belongs to one of the four classes. You also have to solve the meta-puzzle of discovering what class of puzzle you are in. In the language of algorithms, the puzzle classification problem forms an outer loop around the puzzle itself. At any instance, the puzzle may switch, making you realize that you are in a different kind of puzzle than you originally thought.

**Fig. 2:**
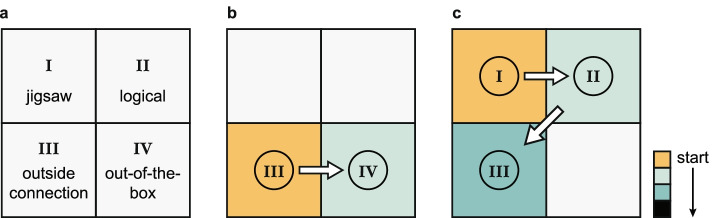
Puzzle switching in research projects. **a** Schematic of the puzzle classes (see Table 1). **b** A switch of puzzle classes in the project studying the gene duplication and alternative splicing. **c** Switches in a deep learning project aiming to predict the substrate scope of enzymes

We give two concrete examples from our own work, where we can pinpoint when switches between puzzle classes occurred. We were interested in gene duplication as a mechanism for generating novel functions and were wondering what aspects could determine the size of a gene family (thus making this a Class III puzzle). This led us to make a connection to alternative splicing: we reasoned that novel isoforms could arise both through gene duplication, as additional gene copies, and through alternative splicing, as additional splice variants [7]. Evidence supporting this connection came in the form of a negative correlation, which suggested that different gene families tended to rely more on one or the other mechanism: families with more gene copies tend to have fewer alternatively spliced isoforms per gene and vice versa. A few years later, however, we realized that there was a deeper reason for this connection: there were two related out-of-the-box tricks that changed the way we saw this relationship [8]. The first trick is considering the gene length, which we found to be correlated with both gene duplication and alternative splicing. The longer a gene is, the more likely it is to generate different splice variants, and the less likely are local genome rearrangements to duplicate the full gene length. The second trick relates to a gene’s expression level. The expression appears to influence both gene duplication and alternative splicing: genes with higher expression levels are less likely to be duplicated and also have more splice variants. When controlling for these two primary gene properties, the correlation between gene duplication and alternative splicing disappears. We needed to reformulate the puzzle from a Class III to a Class IV puzzle (Fig. 2b) to gain a deeper insight into the nature of the relationship between gene duplication and alternative splicing. Only by shifting our focus from the two processes themselves to other gene properties—by thinking outside the box—were we able to understand the observed correlation.

The second example is a project that uses deep learning to predict the substrate scope of enzymes [9]. Our initial idea was to simply modify a prediction pipeline that we had developed previously for the Michaelis constants of enzymes, *K*_*m*_ [10]. We hence started out with what we believed to be a typical Class I problem. Soon after, we realized that we had way too few negative examples—substrates *not* bound by a given enzyme. We had to create random negative examples, but these had to be chosen in a way that helped the resulting model to achieve maximal accuracy. This task required deeper insights into the problem structure, turning it into a Class II problem. But even then, the accuracy of the predictions was relatively low. Converting the problem into Class III, we searched for inspiration by studying methods of natural language processing—the source of many AI methods used for biological problems. We indeed found a solution: we added another “layer” to an existing numerical representation [11] of amino acid sequences. Training this model on our enzyme data, we could teach it to produce output that was more informative for our predictions [9].

It is interesting to consider that there may be typical patterns according to which puzzles switch classes. We ourselves often start a new project with an optimistic class I mindset, seeing it as a jigsaw-like puzzle. We assume that all model components and their potential connections are straightforward, and all that needs to be done is to put them together in the correct arrangement. More often than not, however, we subsequently discover obstacles that force us to follow unforeseen connections to other phenomena (class III), to dive into deeper logical or mathematical problems (Class II), or to identify wrong assumptions that we had initially not questioned (Class IV).

Not knowing what puzzle we are in highlights the uncertainty inherent in any research project. A downside of this uncertainty is the psychological strain it can cause. Adopting the mindset of a puzzle solver may help us to reframe this uncertainty—we may view it as part of a playful process, allowing us to have an open mind and to not stick rigidly to the project’s original framing. Without this playful, puzzle-solving attitude, we may not only limit the joy of doing science. We may also miss out on quite a few insights, big or small.
